# Applying Machine Learning Techniques to Identify Undiagnosed Patients with Exocrine Pancreatic Insufficiency

**DOI:** 10.36469/9727

**Published:** 2019-02-14

**Authors:** Bruce Pyenson, Maggie Alston, Jeffrey Gomberg, Feng Han, Nikhil Khandelwal, Motoharu Dei, Monica Son, Jaime Vora

**Affiliations:** 1 Milliman, New York, NY; 2 AIBrainTree, LLC, Chicago, IL; 3 AbbVie Inc, North Chicago, IL

**Keywords:** predictive modeling, identifying/predicting undiagnosed epi, exocrine pancreatic insufficiency (epi), claims data analysis, case-finding technique, machine learning

## Abstract

**Background:** Exocrine pancreatic insufficiency (EPI) is a serious condition characterized by a lack of functional exocrine pancreatic enzymes and the resultant inability to properly digest nutrients. EPI can be caused by a variety of disorders, including chronic pancreatitis, pancreatic cancer, and celiac disease. EPI remains underdiagnosed because of the nonspecific nature of clinical symptoms, lack of an ideal diagnostic test, and the inability to easily identify affected patients using administrative claims data.

**Objectives:** To develop a machine learning model that identifies patients in a commercial medical claims database who likely have EPI but are undiagnosed.

**Methods:** A machine learning algorithm was developed in Scikit-learn, a Python module. The study population, selected from the 2014 Truven MarketScan® Commercial Claims Database, consisted of patients with EPI-prone conditions. Patients were labeled with 290 condition category flags and split into actual positive EPI cases, actual negative EPI cases, and unlabeled cases. The study population was then randomly divided into a training subset and a testing subset. The training subset was used to determine the performance metrics of 27 models and to select the highest performing model, and the testing subset was used to evaluate performance of the best machine learning model.

**Results:** The study population consisted of 2088 actual positive EPI cases, 1077 actual negative EPI cases, and 437 530 unlabeled cases. In the best performing model, the precision, recall, and accuracy were 0.91, 0.80, and 0.86, respectively. The best-performing model estimated that the number of patients likely to have EPI was about 12 times the number of patients directly identified as EPI-positive through a claims analysis in the study population. The most important features in assigning EPI probability were the presence or absence of diagnosis codes related to pancreatic and digestive conditions.

**Conclusions:** Machine learning techniques demonstrated high predictive power in identifying patients with EPI and could facilitate an enhanced understanding of its etiology and help to identify patients for possible diagnosis and treatment.

## Background

Exocrine pancreatic insufficiency (EPI) is a serious condition characterized by a lack of functional exocrine pancreatic enzymes and the resultant inability to properly digest fats, carbohydrates, and proteins.^1^ Common pancreatic causes of EPI include chronic pancreatitis, severe acute pancreatitis, pancreatic cancer, pancreatic surgery, and cystic fibrosis.^1–3^ Non-pancreatic causes of EPI include a history of celiac disease, diabetes mellitus, Crohn’s disease, gastric surgery, short bowel syndrome, and Zollinger-Ellison syndrome.^1,3^ The main symptoms of EPI are steatorrhea (ie, excess fat in the stool), abdominal bloating/discomfort, and weight loss.^1^

Although an early and accurate diagnosis of EPI is critically important to optimize patient outcomes,^3,4^ the condition remains underdiagnosed.^1^ In addition, there is a lack of consensus regarding the best diagnostic approach, and experts have noted the non-reliability and non-specificity of available diagnostic tests.^1^ Currently available diagnostic tests for EPI include fecal fat quantification, the fecal elastase-1 test, and the^5^ C-mixed triglyceride breath test.^4^ Diagnosing EPI is challenging because its symptoms may be vague or overlap with those of other gastrointestinal disorders.^1^ Other barriers to the diagnosis of EPI have also been identified: (1) currently available tests are cumbersome and unpleasant;^6^ (2) tests are not widely available or accurate for patients in the early to moderate stages of EPI;^6^ (3) there is no International Classification of Diseases, Ninth Revision, Clinical Modification (ICD-9-CM) or Current Procedural Terminology (CPT) code that definitively identifies a patient with EPI, thus tracking has been difficult from a claims perspective;^7^ and (4) use of an International Classification of Diseases, Tenth Revision (ICD-10) code introduced for EPI in October 2016 may not yet be integrated into administrative claims data or electronic medical records (EMR).

The precise incidence and prevalence of EPI are difficult to determine due to its underdiagnosis; furthermore, medical statistics on EPI are usually not reported.^1^ Some data suggest, however, that the prevalence of EPI in patients with chronic pancreatitis is 30% to 40% and that the prevalence of EPI in patients with cystic fibrosis is 80% to 90%.^2^ Studies have also shown that more than 40% of patients with type 1 diabetes mellitus and 30% of patients with type 2 diabetes develop mild to moderate EPI.^2^ At present, reliable estimates of EPI prevalence in the general population are lacking.^4^

Left untreated, EPI can have a deleterious effect on quality of life and may lead to the development of nutritional deficiencies and subsequent malnutrition-related conditions.^4^ The goals of EPI treatment are to alleviate the unpleasant clinical symptoms related to maldigestion and to correct nutritional deficiencies.^8^ Currently, EPI is treated with lifestyle modifications (ie, smoking cessation; alcohol abstinence; and the consumption of frequent, low-volume meals) and oral pancreatic enzyme replacement therapy (PERT), the latter of which is the cornerstone of EPI treatment.^4^ Guidelines established by international societies are largely in agreement that more aggressive treatment of EPI is needed.^6,9–13^

The purpose of the current study was to identify patients in a commercial medical claims database who likely had EPI but were undiagnosed. To that end, we performed a claims data analysis using machine learning, a data analytics approach that is being increasingly used as a predictive tool in the field of medicine.^5,14–24^

## Methods

### Data Source

We used the 2014 Truven MarketScan® Commercial Claims Database (hereafter MarketScan) to identify the study population. MarketScan is a large dataset that contains enrollment data and health benefit claims data for more than 39 million commercially insured lives from more than 100 employer and health plan contributors. The enrollment data include beneficiary age, dependent status, and monthly eligibility status. The claims data include coding information (diagnosis, procedure, revenue, diagnosis-related group, and national drug codes), service dates, and site of service.

### Study Population

The source population consisted of commercially insured individuals selected from the MarketScan database who were 0-64 years old at the end of 2014 and were active or disabled (not part-time or temporary) employees or their dependents. In addition, individuals were required to have prescription drug coverage for the entire duration of their medical coverage period and could not be enrolled in capitated health plans.

For modeling, we extracted all claims from the source data and constructed an enriched study population by selecting individuals who fell into broad categories of diagnoses that are prone to EPI. These categories included inflammatory conditions of the pancreas, other pancreatic conditions (including unspecified diseases of the pancreas), malabsorption syndromes, inflammatory bowel disease, insulin-taking diabetes, and HIV; individuals who had undergone bariatric surgery were also included. We excluded individuals who had conditions that were almost certainly associated with EPI (ie, cystic fibrosis and pancreatic cancer) and those who had undergone radical pancreatic surgeries (ie, pancreaticoduodenectomy, radical subtotal pancreatectomy, or total pancreatectomy). It is highly likely that the excluded individuals would be recognized as being at high risk of EPI, thus we did not want their characteristics to overshadow the characteristics of less EPI-prone cohorts. Non-insulin-dependent diabetics were also excluded from the study population after preliminary data analyses found a low association of EPI in that group. See Table A1 in the Technical Appendix for a full list of the conditions used to include and exclude patients from the study population.

### Machine Learning Algorithm

Machine learning is a computer-based data analytics approach that automates model building through the use of algorithms that iteratively learn from and adapt to subject data.^25^ To develop our models, we used the open source library Scikit-learn, a Python module featuring machine learning algorithms.^26^

Machine learning uses terminology that differs from that of statistical analysis, the latter of which may be more familiar to the health care readership. A basic knowledge of machine learning terminology, however, is needed to understand the methodology used in our analysis. See Table 1 for a comparison of the terminologies used in machine learning and statistical analysis.

Machine learning often uses highly technical iterative processes and trials to develop workable models. A common practice in machine learning is to explore several approaches to improve model fit.

We used a three-step procedure to create the main input to our machine learning model.

1. Two hundred and ninety condition category flags (features) were constructed. These categories were composed of broad, clinically-related ranges of various codes: 74 ICD-9-CM diagnosis code categories, 5 ICD-9-CM procedure code categories, 37 CPT code categories, 70 revenue code (REVCODE) categories, and 104 national drug code (NDC) categories.
2. Each claim for each member of the study population was assigned a flag for each condition category to indicate whether the condition was present (flag = 1) or not present (flag = 0).
3. Claim-level flags were summarized to create a single set of the 290 condition category flags for each member in the study population. The value in each condition category indicated the total number of claims in that category. Patient age and sex, as well as prescription counts for patients who had been treated with a PERT, were also used to create the machine learning model.

We used a nine-step procedure to create our machine learning model and to generate, validate, and assess the results.

1. The study population was divided into three groups: actual positive cases (patients believed to have EPI), actual negative cases (patients believed to not have EPI), and unlabeled cases (patients with unknown EPI status). Actual positive cases (or “true positives”) included patients who had filled ≥ 3 prescriptions for a PERT. Actual negative cases (or “true negatives”) included patients who had undergone a fecal elastase-1 test (CPT code 82656) but had not filled a PERT prescription.
2. The study population was randomly divided into an 80% training subset and a 20% testing subset. The training subset was used to determine the performance metrics and select the highest performing model, and the testing subset was used to evaluate performance of the best-performing machine learning model using data not used to train or select the model.
3. A baseline model was created to predict the classification of an observation based on several input variables – in this case, to classify whether a patient has or does not have EPI. The latter was accomplished using least absolute shrinkage and selection operator (LASSO) with logistic regression, a method that is commonly used to classify data.^27^ LASSO penalizes the absolute size of the regression coefficients, which in practice, leads to sparse coefficients of the input variables in the logistic regression and is helpful when there are many correlated input variables.
4. Additional models were created using other machine learning techniques with different underlying frameworks to determine which framework was best for the input data and model goals. The additional machine learning techniques included gradient-boosted classification trees, support vector machines, and random forest. Random forest is an ensemble method that leverages a collection of decision trees that generate a response when presented with a set of features.
5. Adjustments were applied to handle “imbalanced data,” which occurs when one or more of the binary classes is underrepresented, and included modifying class weights, oversampling, and undersampling. See Table A2 in the Technical Appendix.
6. A 3x3 nested cross validation process was applied to several random forest models to determine the hyperparameters (ie, the set of parameters that defines the properties of the model, such as the number of decision trees in a random forest) and to generate performance metrics. This process was applied to the training subset and consisted of two steps: outer cross validation and inner cross validation. In the outer cross validation step, the training subset was split into three folds (groups). Two of the folds were used to train the model on the parameters, and the remaining fold acted as the validation set. This process was repeated until every fold acted as the validation set one time. Within the training folds assigned by the outer cross validation step, the folds were further split into three sub-folds for the inner cross validation process. Two of the sub-folds were used to tune the parameters, and the remaining sub-fold acted as the validation set. The hyperparameters were optimized by selecting the best performing set, as measured by the average performance metrics over three validation sets through inner cross validation. The performance metrics of the selected random forest models were then calculated in the validation set through outer cross validation. The inner cross validation process was repeated separately over the three outer cross validation splits; that is, we had three sets of hyperparameters from three inner loops and three sets of performance metrics from three outer loops. The performance metrics were averaged for the purpose of choosing the best-performing model.^28^ See Tables A3 and A4 in the Technical Appendix.
7. The performance metrics of 27 models were examined. Model performance was evaluated using each patient’s outputted EPI probability. The metrics used to optimize hyperparameters or to compare the baseline model with other models included precision, recall, F_1_ score, F_beta=10_ score, positive-unlabeled (PU) score, and Brier score loss; see Table 2.
8. The best-performing model was retrained on the full 80% training subset and applied to the 20% testing subset to calculate the performance metrics. Results were summarized in a confusion matrix, which is a commonly used template for presenting the results of binary classifications in machine learning. A receiver-operator characteristic curve was developed.
9. After assigning the probability of EPI, we further examined patients’ claims to determine whether the probability assignments were clinically reasonable. To that end, we used two approaches. In the first approach, we extracted cohorts of patients in the 10% to 20% EPI probability bucket and the 80% to 90% EPI probability bucket and summarized the top 30 code counts for patients in each bucket. In the second approach, we generated a relative importance measure for each feature based on its unique impact on the overall Gini impurity of the model. Gini impurity is a measure of the randomness of the model being evaluated, and the sum of the relative Gini impurity measures for all features is 1.00. Our goal was to minimize the Gini impurity of the model in order to optimize the accuracy of patient classification.

**Table 1.** Terminology Used in Machine Learning and Statistical Analysis

**Machine Learning**	**Statistical Analysis**
Feature	Explanatory variable
Confusion matrix	Contingency table of predicted and actual status
Recall	Sensitivity
Precision	Positive predictive value
F1 score	Harmonic mean of sensitivity and positive predictive value

**Table 2.** Performance Metrics Definitions

**Metric**	**Definition**
Precision	Measure of model exactness; the ratio of successful model predictions over all cases predicted to be positive
Recall	Measure of model completeness; the ratio of successful model predictions over all cases that are actual positives
F_1_ score	Measure of model accuracy; the harmonic mean of precision and recall (reciprocal of the mean of the reciprocals of precision and recall)
F_beta=10_ score	Similar to F_1_ score with an additional parameter (beta) that assigns greater weight to the recall measure of the model
Positive-unlabeled score	Measure of model performance that is positively correlated with the F_1_ score
Brier score loss	Measure of the mean squared differences of the outcome and predictive probability

## Results

In total, 440 695 patients out of the 39 million patients in the MarketScan database met the study inclusion criteria. Of these patients, 2088 were actual positive EPI cases and 1077 were actual negative EPI cases. The remaining 437 530 patients were unlabeled cases. Because actual positives and actual negatives were underrepresented, the data was considered imbalanced.

We found that the three models with the highest performance scores were all random forest models. The baseline LASSO model demonstrated the highest performance in recall, Brier score loss by labeled data, and F_1_ score; however, the baseline model was not the highest performing model overall because it generated an unreasonably high count of positive cases in the unlabeled data (0.93). Three random forest models, which assumed that unlabeled data represented negative EPI cases, generated high recall and precision on the labeled

data. These models also accurately estimated a small number (between 0.04 and 0.06) of positive cases in the unlabeled data and a small Brier score loss (between 0.14 and 0.16) on the full dataset, thereby making them high-performing models. See Table 3.

**Table 3.** Results of Baseline and High-performing Models

	**Baseline**	**1**	**2**	**3**
**Model Type**	**LASSO**	**Random Forest**	**Random Forest**	**Random Forest**
**Metrics**				
Unlabeled data	Ignored unlabeled data	Assumed negative, ignored actual negative cases	Assumed negative, ignored actual negative cases	Assumed negative, ignored actual negative cases
Imbalanced data	N/A	Downsample, class weight	Downsample, subsample balanced weight	Repeated random subsampling
Validation method	80% / 20% split validation	Nested cross validation	Nested cross validation	Nested cross validation
Optimized metric in hyperparameter selection	None	F_(beta=10)_ using 100 random iterations	F_(beta=10)_ × 100 + PU score using 100 random iterations	F_(beta=10)_ × 100 + PU score using 60 random iterations
**Scores**				
F_beta=10_ score (all data)	0.32	0.71	0.71	0.72
PU score (all data)	0.93	9.22	12.45	10.69
Recall (labeled data)	0.92	0.81	0.77	0.80
Brier score loss (labeled data)	0.10	0.14	0.16	0.15
Brier score loss (unlabeled data assumed negative)	0.60	0.06	0.03	0.04
F_1_ score (labeled data)	0.90	0.86	0.84	0.86
Precision (labeled data)	0.88	0.91	0.93	0.91
Probability of unlabeled cases to be labeled as positive	0.93	0.07	0.04	0.06

LASSO: least absolute shrinkage and selection operator; PU: positive-unlabeled

For Model 3, the best performing of the four models, we determined that there were 336 true-positive patients (ie, patients who were accurately predicted to have EPI) and 183 true-negative patients (ie, patients who were accurately predicted to not have EPI), with predicted EPI defined as a patient with an EPI risk of ≥50%. There were 32 false-positive patients (type I error); these were patients who were predicted to have EPI but did not actually have it. There were 82 false-negative patients (type II error); these were patients who had EPI but were not predicted to have it. See Table 4, which shows the confusion matrix for the 88,140 patients in the testing subset.

**Table 4.** Confusion Matrix of Model -3

		**Predicted Condition**		
		**No EPI**	**EPI**	
**Actual Condition**	**No EPI**	183 (True negatives)	32 (False positives, type I error)	
	**EPI**	82 (False negatives, type II error)	336 (True positives)	
	**Unknown**	82 546	4961	

Note: The 2x2 cells shaded in gray represent the confusion matrix

EPI: exocrine pancreatic insufficiency

The number of patients known to have EPI in the testing subset (82 false negatives + 336 true positives) was 418. Of the 87 507 previously unlabeled patients in the testing subset, 4961 patients (6%) were identified as being likely to have EPI, which is approximately 12 times the number of patients in the study population who were identified as being positive for EPI.

From the confusion matrix, we determined key evaluation metrics pertaining to the performance of Model 3, including precision, recall, and F_1_ score. Precision, the ratio of true positives over the sum of true positives and false positives (336/{32+336}), is a measure of exactness and was found to be 0.91. Recall, the ratio of true positives over the sum of true positives and false negatives (336/{82+336}), quantifies the completeness of model results and was found to be 0.80. The F_1_ score, the harmonic mean of precision and recall, depicted as the reciprocal of the mean of the reciprocals of precision and recall ({0.91×0.80}/{0.91+0.80}×2), is a measure of accuracy that was found to be 0.86, where 1.0 represents perfect accuracy. The area under the receiver-operator characteristic curve (AUROC) is a measure of the predictability of the model. An AUROC equal to 1.00 represents a model that perfectly predicts an outcome. An AUROC of 0.50 represents a model with no predictability. In our study, AUROC for the labeled data was found to be 0.94.

In the final step of our study, we applied Model 3 to the 20% testing subset, which assigned each patient a probability of having EPI. We observed that out of a sample of 88 140 patients, 5329 patients (6%) were now captured as EPI patients, with EPI defined as a patient with an EPI risk of ≥ 50%. If the EPI risk threshold was increased to 75%, only 1376 patients (2%) were captured as EPI patients. See Table 5.

In practice, Model 3 could be further applied to the training subset or to new datasets to identify potential previously unidentified patients with EPI. It is important to note, however, that model performance will vary when applying any model to new datasets that differ significantly from the 80% training subset (eg, in terms of population demographics or other characteristics). In such situations, the results should be interpreted with caution, and if possible, model performance should be re-evaluated on the new population and data source.

From our lower (10% to 20%) and upper (80% to 90%) tail analysis for clinical reasonability, we observed that two CPT code features – Evaluation and Management (E&M) (99201 – 99499) and Pathology (80047 – 89398) – were the forerunners in both probability buckets, where nearly all patients had at least one E&M or Pathology claim. The finding was not unexpected, as the study population represented an enriched group of patients with pancreatic conditions that would require laboratory work and diagnostic or management evaluations. These two features, therefore, were not key predictors in determining EPI probability. We observed several other overlapping features between the 10% and 20% EPI probability bucket and the 80% to 90% EPI probability bucket, such as Mental (diagnoses) and Radiology – diagnostic (procedures); however, in the 80% to 90% EPI probability bucket, more features related to conditions of the pancreas and digestive system were found at the top of the list than was the case in the 10% to 20% EPI probability bucket. See Tables 6a and 6b.

**Table 5.** Probability of EPI Assigned to Each Patient by Model 3

**Probability of EPI**	**Number of Patients**	**Predicted to have EPI**
0% - < 5%	35 868	Not likely to have EPI
5% - < 10%	18 022	
10% - < 15%	11 895	
15% - < 20%	7470	
20% - < 25%	4186	
25% - < 30%	2248	Possibly likely to have EPI
30% - < 35%	1085	
35% - < 40%	711	
40% - < 45%	671	
45% - < 50%	655	
50% - < 55%	719	Likely to have EPI
55% - < 60%	666	
60% - < 65%	678	
65% - < 70%	884	
70% - < 75%	1006	
75% - < 80%	767	Highly likely to have EPI
80% - < 85%	397	
85% - < 90%	192	
90% - < 95%	20	
95% - 100%	0	
**Total**		
0% - < 25%	77 441	
25% - < 50%	5370	
50% - < 75%	3953	
75% - < 100%	1376	

EPI: exocrine pancreatic insufficiency

**Table 6a.** Most Frequent Features in Patients with 10% to 20% EPI Probability

**Feature**	**Code Type**	**Portion of Patients with ≥1 Observations**	**Average Number of Observations for Patients with ≥1 Observations**
Evaluation and Management	CPT	100%	15.81
Pathology	CPT	97%	28.03
Medicine	CPT	90%	17.72
Special Encounters	DIAG	78%	24.07
Other Symptoms	DIAG	77%	21.96
Radiology	CPT	74%	5.85
Cardiovascular	CPT	73%	3.84
Metabolic	DIAG	69%	24.45
Musculoskeletal	DIAG	64%	24.15
Laboratory	REVCODE	62%	16.59
Diabetes	DIAG	56%	41.46
Hypertensive	DIAG	56%	23.94
Ulcer Drugs	NDC	55%	4.96
Other Analgesics	NDC	55%	4.56
Antibiotics	NDC	55%	2.22
Respiratory	DIAG	54%	14.80
Other Special Encounters	DIAG	52%	7.98
Insulin	NDC	50%	7.08
Genitourinary	DIAG	49%	25.70
Antihypertensives	NDC	46%	6.43
Digestive	DIAG	45%	15.10
Antihyperlipidemics	NDC	44%	6.39
Pharmacy	REVCODE	43%	4.48
Labs Vitamin Levels Test	CPT	43%	2.56
Anesthesia	CPT	42%	1.93
Mental	DIAG	42%	17.15
Esophagus	DIAG	40%	12.62
Radiology – Diagnostic	REVCODE	40%	2.07
Antidepressants	NDC	39%	6.96
Drugs Requiring Specific Identification	REVCODE	38%	6.54

CPT: current procedural terminology; DIAG: diagnosis; EPI: exocrine pancreatic insufficiency; NDC: national drug code; REVCODE: revenue code

Note: Observations were tabulated at the claim level

**Table 6b.** Most Frequent Features in Patients with 80% to 90% EPI Probability

**Feature**	**Code Type**	**Portion of Patients With ≥1 Observations**	**Average Number of Observations for Patients With ≥1 Observations**
Evaluation and Management	CPT	100%	23.03
Pathology	CPT	96%	35.25
Medicine	CPT	83%	15.11
Other Pancreatic Conditions	DIAG	82%	14.50
Inflammatory Conditions Of Pancreas	DIAG	81%	44.52
Special Encounters	DIAG	76%	34.87
Other Symptoms	DIAG	76%	35.14
Cardiovascular	CPT	73%	4.35
Ulcer Drugs	NDC	72%	6.03
Radiology	CPT	69%	6.53
Other Analgesics	NDC	67%	10.23
Symptoms (Abdominal And Pelvis)	DIAG	65%	49.05
Laboratory	REVCODE	65%	23.98
Musculoskeletal	DIAG	63%	23.38
Digestive	DIAG	58%	42.34
Metabolic	DIAG	58%	33.28
Radiology Abdominal	CPT	57%	4.77
Esophagus	DIAG	55%	23.38
Mental	DIAG	53%	34.26
Antibiotics	NDC	53%	2.25
Other Special Encounters	DIAG	53%	9.54
Respiratory	DIAG	52%	26.16
Pharmacy	REVCODE	50%	9.30
Digestive Surgery	CPT	50%	4.90
Hypertensive	DIAG	50%	34.33
Anesthesia	CPT	49%	2.82
Genitourinary	DIAG	48%	26.69
Emergency Room	REVCODE	47%	5.09
Antidepressants	NDC	45%	7.59
Drugs Requiring Specific Identification	REVCODE	44%	8.47

CPT: current procedural terminology; DIAG: diagnosis; EPI: exocrine pancreatic insufficiency; NDC: national drug code; REVCODE: revenue code

Note: Observations were tabulated at the claim level

From our analysis for Model 3 of the impact of each feature on Gini impurity, we found that two features indicating pancreatic diagnoses were the most important determinates in assigning EPI probability. Table 7 summarizes the top 30 features, which accounted for 90% of the model’s decrease in Gini impurity.

Using the Gini impurity approach, we found that among patients with low EPI probability, the absence of diagnosis codes related to conditions of the pancreas and digestive system was the feature that contributed most to low-probability assignments. For example, a randomly selected patient who was assigned a <10% probability of EPI did not have any diagnosis codes related to inflammatory conditions of the pancreas, other pancreatic conditions, or malabsorption syndromes, and was a younger patient (age 37 years). Conversely, a randomly selected patient who was assigned a >90% probability of EPI had been diagnosed with pancreatic conditions and malabsorption syndromes, was an older patient (age 55 years) and had received prescriptions for ulcer drugs.

**Table 7.**
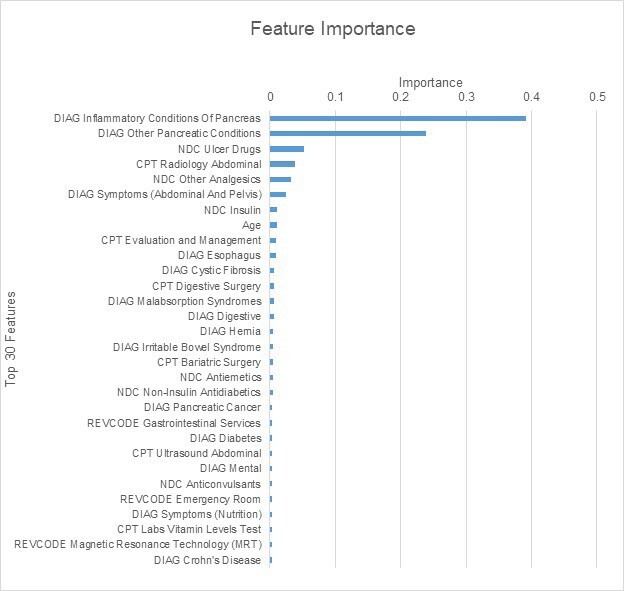
Summary of Model 3 Feature Importance as Measured by Each Feature’s Contribution to Decrease in Gini Impurity CPT: current procedural terminology; DIAG: diagnosis; NDC: national drug code; REVCODE: revenue code Note: The sum of all features’ contributions to the decrease in Gini impurity = 1.00

## Discussion

EPI is a serious condition that remains underdiagnosed because of the nonspecific nature of clinical symptoms, lack of an ideal diagnostic test, and the inability to easily identify patients with EPI using administrative data. We performed an analysis using machine learning techniques to identify patients in the MarketScan database who likely had EPI but had not been diagnosed with the condition. For the study population, we identified 12 likely EPI patients for every actual EPI patient, a finding that suggests a successful machine learning model for identifying likely undiagnosed patients despite current clinical and administrative barriers.

Machine learning is still in its infancy, thus studies evaluating its value and applicability have understandably focused mainly on technical methodology, with leaders in the field sharing analytic techniques. In recent years, however, numerous studies have described clinical applications of machine learning that might be accessible to healthcare decision-makers. For example, machine learning techniques have been used to predict a wide range of events and outcomes in medicine, including early hospital readmissions,^5^ laboratory test results,^14^ treatment requirements,^21^ treatment success,^15,22^ disease progression,^18^ disease complications,^20^ posttraumatic stress,^19^ suicide risk,^16^ graft failure after transplantation,^17^ optimal drug dosage,^29^ and adverse drug reactions.^24^ Most notably, the use of machine learning techniques has garnered much attention in several major therapeutic areas, including diabetes,^20,30^ cancer,^31^ cardiology,^32^ ophthalmology,^21,33^ and psychiatry.^15,34,35^ Two recent studies utilized machine learning in particularly novel ways – in one case, to evaluate the trend in sentiment toward papillomavirus vaccination using Twitter data,^36^ and in the other case, to predict swine movements within a regional program to improve the control of infectious diseases in the US.^37^

The use of machine learning offers practical solutions to real-world challenges in healthcare, even those subject to the limitations of real-world data. Our findings showed that from a public health or market perspective, machine learning is a potentially useful tool to estimate the prevalence of EPI, a condition known to be underdiagnosed. Indeed, the case-finding technique used in our study could be applied to other conditions that may be frequently undiagnosed or nearly impossible to identify in administrative claims data. It is important to note that administrative claims data, which are often used in real-world analytics, are readily available on the scale of tens of millions of lives across multiple years and from virtually all sites of care. A challenging future research endeavor would be to validate our model using EMR, a data source that is notoriously difficult to use on a large-scale basis. Although EMR data provide details of patient complaints and diagnostic values, several barriers to its use should be recognized: the data are complex, patient information may be missing due to the use of multiple providers who are not in the same system, and the consolidation and assemblage of information from different systems and multiple sites pose administrative challenges.

Most of the models we tested in our study performed relatively well; however, we anticipate that additional machine learning and statistical methods used in future studies could potentially improve our probability estimates. In fact, recent studies have examined estimating class priors in unlabeled data and improving models being trained primarily on positive and unlabeled data.^38,39^ To further improve the metrics, a greater number of features – beyond the 290 used in our study – could be added to the inputs to refine the current EPI model, or additional populations could be included to profile for variant EPI subpopulations with mild to moderate pancreatic dysfunction (eg, non-insulin-dependent type 2 diabetics); examples include time between procedures, variables from previous years, and impressions of causal or correlated conditions or treatments from clinical experts. Additional validation of the model’s predictability could be achieved by following patients for several years and observing who started PERT therapy, or by mining EMR to look for an EPI diagnosis. Finally, an ICD-10 code for EPI, K86.81, was introduced in October 2016, thus the models used in our study could be revisited to use an actual EPI diagnosis code to identify EPI patients, in addition to using treatment with PERT to indicate actual positives for EPI.

We believe that machine learning models can be practical, cost-effective tools for organizations with the necessary resources, which include strong medical claims database capabilities and knowledge of medicine, actuarial science, and statistics. By way of example, our team consisted of about a dozen individuals with diverse backgrounds, including actuarial, statistical, clinical, and healthcare data analytics, all of whom worked substantially less than fulltime and largely completed the analysis within three months.

We acknowledge several limitations in our study. First, although we used the standard approach of randomizing data into training and testing subsets, we could not verify the findings through chart audits. We regard the latter as an important future step and envision that an insurer could validate these findings through its case management efforts and further train the model to increase its accuracy. Second, the resources dedicated to running the models – both time and computer resources – could be constraints for organizations trying to apply our approaches. A small insurer, for example, may not have the resources to evaluate or implement the model. Third, the usual limitations of administrative claims data also applied to our study. Claims data, although comprehensive and adequate in many ways, are subject to variation in provider coding practices and inaccuracies. The latter limitation, however, is not unique to our study, but rather reflects the status of real-world data that any organization implementing a case-finding technique would encounter.

## Conclusions

Administrative claims data, although readily available on the scale of tens of millions of lives, lack many of the clinical details that can be found in EMR; however, obtaining large-scale EMR data has proved to be difficult. Machine learning approaches applied to administrative claims data offer a fast and practical approach to researching important healthcare challenges.

The high predictive power of our EPI model shows that applying machine learning techniques to administrative claims data can offer practical and efficient solutions to understanding real-world healthcare challenges. Although definitive claims about the implications of our findings on clinical practice cannot be made, we submit that our study has demonstrated the feasibility and potential value of using machine learning as an efficient strategy for predicting EPI in undiagnosed patients and perhaps will inspire future researchers to improve our probability estimates and extend our findings.

## Disclosure

Funding for this work was provided to Milliman, Inc. by AbbVie Inc.

## Acknowledgments

We would like to thank Kathleen Wildasin for her very helpful editorial support. We would also like to thank Marjorie Schulman, MD; Jason Tsai, MD, MS of AbbVie Inc.; as well as Shea Parkes, FSA; Christine Ferro; Sean Pittinger; Mona Kelkar; and Melissa Caplen of Milliman, Inc. for their technical support. Finally, we would like to thank Rachelle Fox of ICON Plc. for her oversight of the approval process.

## Supplementary Material

Supplementary Content
