# Supplementary material for: Applying Machine Learning Techniques to Identify Undiagnosed Patients with Exocrine Pancreatic Insufficiency
Source: J Health Econ Outcomes Res. 2019 Feb 14;6(2):32–46. doi: 10.36469/9727 (PMC7299452; doi:10.36469/9727)
Supplement: Supplementary Content [file jheor_2019_6_2_9727_34289.pdf]

JHEOR Journal of Health Economics and Outcomes Research

OPEN ACCESS

# Supplementary Online Content

Pyenson B, Alston M, Gomberg J, *et al.* Applying machine learning techniques to identify undiagnosed atients with exocrine pancreatic insufficiency. *JHEOR*. 2019;6(2):32-46.

**Technical Appendix**
**Table A1.** Hierarchy of EPI-related Conditions
**Table A2.** Treatment of Imbalanced Data
**Table A3.** Treatment of Unlabeled Data
**Table A4.** Approaches Used to Validate Models

This supplementary material has been provided by the authors to give readers additional information about their work.

## Technical Appendix

### *Python Code*

The machine learning algorithms produced for the Baseline model and Models 1-3 can be found at: https://github.com/milliman/EPI_Machine_Learning "Applying Machine Learning Techniques to Identify Undiagnosed Patients with Exocrine Pancreatic Insufficiency".

### *Hierarchy of EPI-related Conditions*

To generate an enriched study population of individuals who were moderately prone to EPI but did not have conditions associated with a very high likelihood of EPI, we constructed a hierarchy of EPI-related conditions; see Table A1. We used this hierarchy to include or exclude individuals from the study population. To be identified with most conditions in the hierarchy, a patient needed to have ≥ 1 acute inpatient or observation claim or ≥ 2 non-acute inpatient, outpatient, Evaluation & Management, or emergency department claims on different dates of service. For some conditions, we used a loosened criterion because the seriousness of these conditions made "rule out" coding unlikely. For the latter conditions, a patient needed to have only one acute inpatient or observation claim or one non-acute inpatient, Evaluation & Management, outpatient, or emergency room claim in order to be flagged with the condition.

### *Methods Used to Address Data Issues*

If we assumed that the unlabeled data were negative, we created imbalanced data. Table A2 presents the approaches we used across the 27 models to treat imbalanced data in our model. Of the six approaches listed, only downsampling and repeated random subsampling were used in the final models.

Table A3 describes the approaches we used to handle unlabeled data.

### *Approaches Used to Validate Models*

We considered three validation approaches in our study; see Table A4. All of these methods partitioned the data into a training subset and a testing subset. Candidate models were developed using the training subset, and the models were compared based on their performance metrics. The best model was applied to the testing subset to produce performance metrics.

## References

[1] Lee WS, Liu B. Learning with positive and unlabeled examples using weighted logistic regression. *Algorithmic Learn Theory* 2003;348(1):71-85.

[2] Khalilia M, Chakraborty S, Popescu M. Predicting disease risks from highly imbalanced data using random forest. *BMC Med Inform Decis Mak* 2011;11(1):51.

[3] Chen C, Liaw A, Breiman L. Using Random Forest to Learn Imbalanced Data. Technical Report 666. Statistics Department of University of California at Berkeley.

[4] Chawla NV, Bowyer KW, Hall LO, et al. SMOTE: synthetic minority over-sampling technique. *J Artif Intelligence Res* 2002;16(1):321-57.

[5] Cawley GC, Talbot NLC. On over-fitting in model selection and subsequent selection bias in performance evaluation. *J Mach Learn Res* 2010;11:2079-2107.

**Table A1.** Hierarchy of EPI-related Conditions

| Rank | Condition | Code |
|---|---|---|
| **1** | **Cystic Fibrosis** | **277.0X** |
| **2** | **Pancreatic Cancer** | **157.0 – 157.9, 230.9** |
| **3** | **Radical Pancreatic Surgery** | |
| **3.1** | **Radical Pancreaticoduodenectomy, Radical Subtotal Pancreatectomy, Total Pancreatectomy** | **52.7; 52.53; 52.6** |
| 3.2 | Partial Pancreatectomies: Proximal, Distal, Not Elsewhere Classified (L) | 52.51; 52.52; 52.59 |
| 3.3 | Other Pancreatic Surgery (L) | 52.2X, 52.3 and 48105 – 48155 (CPT) |
| 3.4 | Other pancreatic neoplasms, not necessarily treated surgically (L) | 211.6, 211.7, 251.8, 251.9 |
| 4 | Inflammatory Conditions of Pancreas: Acute Pancreatitis, Chronic Pancreatitis (L) | 577.0; 577.1 |
| 5 | Other Pancreatic Conditions (L) | 577.8, 577.9 |
| 6 | Malabsorption Syndromes: Celiac Disease, Tropical Sprue, Other Malabsorption Syndromes, Whipple's Disease | 579.0; 579.1; 579.2 – 579.9; 040.2 |
| 7 | Bariatric Surgery: Laparoscopic and Bariatric Surgery, Open | 43644 – 43648, 43770 – 43775 (CPT); 43842 – 43846, 43886 – 43888 (CPT) |
| 8 | Inflammatory Bowel Diseases | |
| 8.1 | Irritable Bowel Syndrome | 564.1 |
| 8.2 | Ulcerative Colitis | 556 |
| 8.3 | Crohn's Disease | 555 |
| 9 | Diabetes | 250.XX |
| 9.1 | Insulin takers | |
| *9.2* | *Non-insulin takers* | |
| 10 | HIV (excluding asymptomatic patients) | 042 |
| *11* | *All other* | |

CPT: current procedural terminology; EPI: exocrine pancreatic insufficiency.
Note: All codes are International Classification of Diseases, Ninth Revision, Clinical Modification codes (ICD-9-CM), unless otherwise specified. Patients with conditions shown in bold font were excluded from the study population, as it is highly likely that affected patients had already been diagnosed with EPI, and we did not want the characteristics of these patients to overshadow the characteristics of less EPI-prone patients. Patients with conditions shown in italicized font were excluded from the model due to their low association with EPI. The loosened criterion was applied to all conditions with an (L) designation.

**Table A2.** Treatment of Imbalanced Data

| Approach | Description | Application in Final Models |
|---|---|---|
| **Downsampling** | This method randomly selected unlabeled data for use as labeled negative to achieve a targeted balance between labeled positive and labeled negative data. Between 2000 and 15 000 unlabeled cases were randomly chosen to be combined with the actual negative cases. | Models 1-2 only |
| **Repeated random subsampling** | This method assigned all unlabeled cases as negative and partitioned them into subsamples with a predetermined ratio per subsample between majority and minority cases. Multiple training subsets using the subsampled negative cases and all positive cases were used to create an ensemble of models. A majority vote of the models was used to determine the output of the ensemble.* | Model 3 only |
| **Class weighting** | This method weights the minority class (eg, positive cases) to be more important relative to the majority class (eg, negative cases). Many machine learning techniques can accept a class weighting scheme to balance the ratio of classes. For example, if there are 100 majority class examples and 10 minority class examples, a "balanced" weight would be 1.0 and 10.0, respectively, which would lead to each class having a total weight of 100.0. | None |
| **Subsample balanced weight** | This method weights the minority class more heavily relative to the majority class, just as the class weighting method does, but separately for every bootstrapped tree in a random forest technique. For example, Tree 1 may have a sample of 90 negatives and 20 positives, so the class weight of the positives is 4.5 and that of the negatives is 1.0. Tree 2 may have a sample of 105 negatives and 5 positives, so the class weight of the positives would be 21.0 and that of the negatives would be 1.0 for that specific tree only. Every tree is independently weighted before being trained based on the bootstrap sample of cases. | None |
| **Bootstrapped downsampling** | This method modifies the normal random forest technique to downsample every bootstrap sample for every decision tree in the random forest to a specified minority to majority class ratio. For example, if there are 100 majority class examples and 10 minority class examples, and the target ratio is 0.5, every tree in the random forest would be assigned a sample of 10 minority class examples with 20 majority class examples. From that subsample, a bootstrapped population of 30 would be chosen to train that specific tree. This process is repeated for every tree in the random forest. The bootstrapped downsampling method is similar to a "balanced random forest." ** | None |
| **Synthetic minority oversampling technique (SMOTE) resampling** | This method assigns all unlabeled cases as negative and resamples positive data to achieve a targeted balance between labeled positive and labeled negative cases. SMOTE resampling attempts to achieve a more distinct classification between positive and negative data.*** | None |

*Repeated random subsampling has been shown to be effective in dealing with imbalanced data in the context of a random forest approach to medical outcomes research.[2]

**A balanced random forest approach balances the positive class and negative class in every tree of the random forest.[3]

***SMOTE is an approach that oversamples cases in the underrepresented class.[4]

**Table A3.** Treatment of Unlabeled Data

| Approach | Description | Application in Final Models |
|---|---|---|
| **Ignored unlabeled data** | All unlabeled data were ignored; only labeled positive and labeled negative data were used during the training of models. | Baseline only |
| **Assumed unlabeled data to be negative and ignored actual negative cases** | All "actual negative" cases were ignored during the training of models; unlabeled data were assumed to be negative. In the literature, this method is often called positive-unlabeled (PU) learning.* | Models 1-3 only |

*PU learning is an alternative approach in which the study population consists primarily of unlabeled and actual positive cases.[1]

**Table A4.** Approaches Used to Validate Models

| Approach | Description | Application in Final Models |
|---|---|---|
| **80/20 Split Validation** | The simplest form of validation is to split the data into a training subset and a testing subset. In our study, we used an 80%/20% ratio of training data to validation data in the baseline model. | Baseline only |
| **Stratified K-Fold Cross Validation** | Using stratified K-fold cross validation, the training subset was is divided into multiple training and validation subsets to estimate how the models would generalize to new unseen data. The data are divided into "K" folds (eg, 3 or 5), whereby each fold is representative of the whole dataset in terms of percentage of cases that are unlabeled, negative, and positive. "K" models with the same hyperparameters are trained, such that, where the training data for each model leaves one fold (ie, the validation fold) out of training so that the metrics are computed on the validation fold after the training is complete. | None |
| **(K x N) Nested Cross Validation*** | (K x N) Nested cross validation is the method used in Models 1-3. (K x N) Nested cross validation consists of two steps: outer cross validation and inner cross validation. In the outer cross validation step, the training subset is split into "K" folds (groups). "K-1" folds are used to train the model on the parameters, and the one remaining fold acts as the validation set. This process is repeated until every fold acts as the validation set one time. Within the training folds assigned by the outer cross validation step, the folds are further split into "N" folds in the inner cross validation process. "N-1" folds in the inner cross validation step are used to tune the parameters, and the one remaining fold acts as the validation set. The hyperparameters are optimized by selecting the best performing set, as measured by the average performance metrics over "N" validation sets through the inner cross validation. The performance metrics of the model are then calculated in the validation set through outer cross validation. The inner cross validation process is repeated separately over the "K" outer cross validation splits. When comparing multiple machine learning model types along with tuning hyperparameters for each technique, using nested cross validation has been shown to produce unbiased, accurate generalization estimates that can be used to responsibly compare models and model types.[5] In our study, we used 3 for both "K" and "N". | Models 1-3 only |

Note: When comparing multiple machine learning model types or tuning hyperparameters, nested cross validation has been shown to produce unbiased, accurate generalization estimates that can be used to responsibly compare models and model types.[5]
